# Psychometric evaluation of a novel tool for assessing gestational diabetes and hypertension care: knowledge, attitudes, and practices of midwives and nurses

**DOI:** 10.25122/jml-2024-0146

**Published:** 2024-02

**Authors:** Daniela Stan, Claudia Elena Dobre, Doina Carmen Mazilu, Elvira Brătilă

**Affiliations:** 1 Department of Obstetrics and Gynecology, Carol Davila University of Medicine and Pharmacy, Bucharest, Romania; 2 Department of General and Specific Nursing, Carol Davila University of Medicine and Pharmacy, Bucharest, Romania

**Keywords:** knowledge, attitudes, practice, midwife, nurse, psychometric qualities, gestational hypertension, gestational diabetes, GD, Gestational Diabetes, HBP, High Blood Pressure, M, Midwives, ON, Obstetric Nurses, KAP, knowledge, attitudes, and practices, PIH, Pregnancy-Induced Hypertension, OGTT, Oral Glucose Tolerance Test, SBP, Systolic Blood Pressure, DTB, Diastolic Blood Pressure, BMI, Body Mass Index, BP, Blood Pressure

## Abstract

While standardized assessment of knowledge, attitudes, and practices (KAP) related to gestational diabetes and hypertension is possible with a valid tool, existing research remains limited. This prospective validation study aimed to develop and validate a novel tool to assess the KAP of midwives and obstetric nurses. We included 125 midwives and obstetric nurses who routinely care for patients with gestational diabetes and hypertension. The tool demonstrated good internal consistency (Cronbach's alpha): knowledge (0.729, 95% CI, 0.654–0.776), attitude (0.756, 95% CI, 0.690–0.814), and practices (0.925, 95% CI, 0.905–0.943). Difficulty indices (d) ranged from 0.38 to 0.99 (knowledge), 0.41 to 0.99 (attitudes), and 0.41 to 0.93 (practices), indicating appropriate item difficulty. Discrimination indices (D) confirmed items could differentiate between respondents with low and high knowledge levels (D range: 0.02–0.77 for knowledge, 0.06–0.64 for attitudes, 0.20–0.84 for practices). The robust psychometric properties of this tool support its use in future research on KAP related to diabetes and gestational hypertension management in midwives and nurses. This instrument has the potential to be valuable in various settings, including baseline assessment before educational programs or evaluation of learning outcomes after interventions.

## INTRODUCTION

High blood pressure (HBP) and gestational diabetes (GD) are two complications frequently encountered during pregnancy with potentially adverse effects on both the mother and the fetus [1]. Studies investigating the association between these medical conditions and pregnancy have shown adverse effects on children born to mothers with HBP (higher rates of early-onset cardiovascular disease) and gestational diabetes (increased newborn adiposity with elevated maternal blood glucose) [2]. In addition, pregnancy-related complications such as hypertensive disorders, GD, preterm birth, and pregnancy loss have been associated with an increased risk of future cardiovascular disease (CVD). This highlights the importance of recognizing and addressing these risk factors during pregnancy to fully assess a woman's future cardiovascular risk and develop appropriate risk reduction strategies [3].

Hypertension in pregnancy affects approximately 5-10% of pregnancies and can have a significant impact on maternal, fetal, and neonatal outcomes. It can be diagnosed through routine blood pressure monitoring during prenatal visits [4,5]. In the second half of pregnancy, HBP may be accompanied by proteinuria and may induce adverse neonatal and maternal outcomes in both adolescent and adult women, increasing the risk of preterm birth among adolescents [6,7]. Gestational diabetes is another common second-trimester complication frequently associated with hypertension, especially in overweight women [8,9]. It has been shown that women with GD have a higher risk of developing hypertensive disorders, especially in the postpartum period [10-12].

Research shows that good glycemic control in patients with GD can reduce the risk of pregnancy-induced hypertension (PIH) and low birth weight with good maternal-fetal care outcomes [13]. In this context, the screening of pregnant patients for early identification of GD and gestational HBP represents essential care to reduce maternal-fetal complications [14]. Given their central role in detecting potential pregnancy complications, midwives require adequate skills for the early detection of both HTA and GD [6].

In Romania, effective management of these conditions by midwives (M) and obstetric nurses (ON) necessitates up-to-date knowledge, attitudes, and practices aligned with the best practices in the field and according to their competencies [15]. M and ON must recognize the early signs of GD and gestational HBP, implement necessary measures to maintain blood glucose values within normal limits, limit excessive weight gain, monitor blood pressure, administer prescribed medication, and educate patients on self-management [15].

Previous studies assessing the KAP of healthcare professionals caring for pregnant women highlight the need for continuous knowledge updates [16-21]. Garti *et al*. [16] revealed that midwives do not have sufficient knowledge to diagnose and provide adequate medical care to pregnant patients with preeclampsia and that the development of policies that focus on the innovative continuous training of this professional category is necessary.Another cross-sectional study conducted in Jakarta on a group of 639 practicing midwives showed that only 50.2% had sufficient knowledge about pregnancy-induced hypertension (PIH), while 58.2% had adequate knowledge about the clinical examination and diagnosis of PIH, and 63% of these had good knowledge about gestational HBP management [17]. Similar results were identified in a UK study of healthcare professionals' knowledge of hypertension management and theoretical notions of blood pressure thresholds [18]. Furthermore, a study evaluating nurses' knowledge of evidence-based practices in preeclampsia care found that over a third of participants demonstrated only intermediate knowledge [19]. A study in Ontario identified barriers and facilitators influencing midwives' care in GD and gestational HBP, emphasizing interdisciplinary collaboration [20]. In Romania, only one study identified and highlighted a limited level of knowledge about preeclampsia and eclampsia among midwives and resident doctors, emphasizing the need for additional training on the early identification and correct management of these conditions [21].

Although several studies have evaluated the levels of KAP related to GD or HBP among healthcare professionals, these studies often lack information regarding the reliability and validity of assessment tools. This study addresses this gap by evaluating the validity and reliability of a new tool specifically designed to assess the KAP of Romanian M and ON regarding GD and HBP management.

## MATERIAL AND METHODS

This prospective instrument validation study aimed to develop a reliable and valid tool specifically tailored to assess the KAP of Romanian M and ON regarding GD and HBP management. This tool can inform targeted educational programs, ultimately improving medical care for pregnant women with GD and HBP.

The development and validation of the assessment tool occurred in two phases. The first phase focused on establishing construct validity. A focus group of 25 experts, including doctors and midwives experienced in GD and HBP care, evaluated the tool. Their feedback ensured its alignment with Romanian M and ON practices and competencies, as well as overall relevance, clarity, and answer choice appropriateness. Focus group members completed the questionnaire, providing valuable feedback and observations for refining wording, item relevance, and accuracy. The second phase assessed the psychometric properties of the questionnaire using various statistical indices, including the difficulty index, discrimination index, and Cronbach’s alpha coefficient.

The final questionnaire comprised 68 items that assessed the level of KAP of M and ON regarding HBP and GD. It included 12 items for demographic data and 56 KAP items distributed across three scales:

**Knowledge scale (15 items):** Single-choice or open-ended questions to assess knowledge about HBP and GD.

**Attitude scale (18 items):** Likert scale with five response options (‘strongly agree’, ‘agree’, ‘neither agree nor disagree’, ‘disagree’, and ‘strongly disagree’) to evaluate perceptions and beliefs towards these conditions.

**Practice scale (23 items):** Measured self-reported behaviors in managing patients with HBP and GD by offering three answer choices (‘always’, ‘sometimes’, and ‘never’) for specific medical care activities.

This study was conducted at the Prof. Panait Sîrbu Clinical Hospital of Obstetrics and Gynecology in Bucharest, Romania. A random sample of 125 M and ON from the obstetrics and gynecology departments participated. The sample size was calculated based on a 95% confidence level (CI), a 6% margin of error, and a target population of 234 M and ON at the hospital. Participants from the focus group, students, and healthcare professionals from other specialties (e.g., neonatology nurses, physiotherapists, psychologists, and doctors) were excluded.

### Statistical analysis

Data analysis was performed using IBM SPSS Statistics 20.0. Descriptive statistics summarized sociodemographic characteristics. Difficulty and discrimination indices and Cronbach’s alpha coefficient were calculated to assess the questionnaire's psychometric properties.

## RESULTS

### Sociodemographic characteristics of the respondents

**Figure 1:**
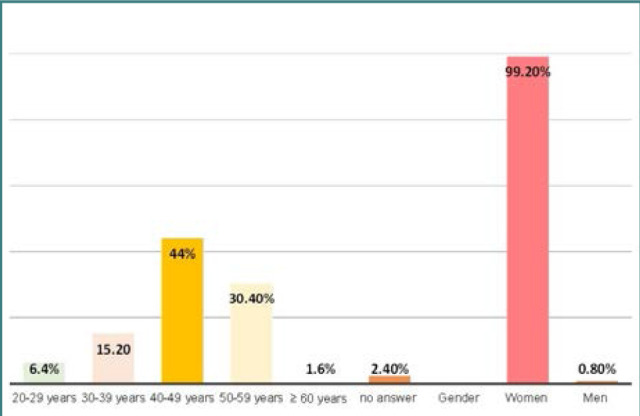
Sociodemographic characteristics of respondents

**Figure 2:**
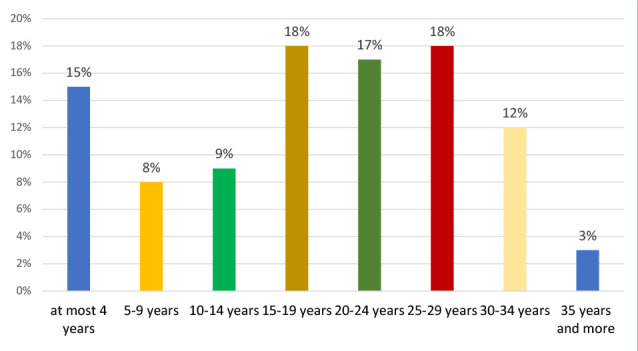
The professional experience of respondents

**Figure 3:**
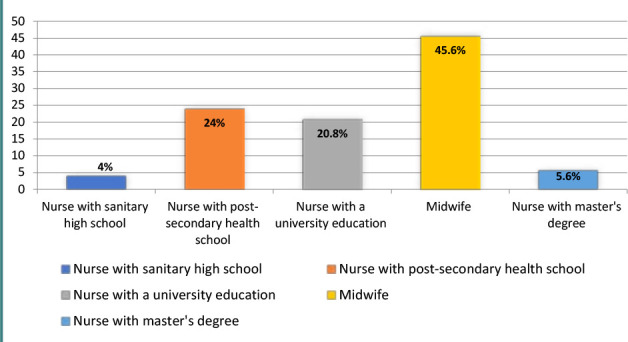
The professional training of respondents

A total of 125 midwives (M) and obstetric nurses (ON) working at Prof. Dr. Panait Sîrbu Hospital of Obstetrics and Gynecology participated in the study. The mean age of participants was 44.66 years (SD = 8.24 years), and the majority were female participants (99.2%) (Figure 1). The average professional experience was 18.2 years (SD = 10.34 years). (Figure 2) Regarding professional training, 72% of participants completed higher-level training (bachelor's or master's degrees), and 28% completed post-secondary professional training (Figure 3).

### Evaluation of difficulty (d) and discrimination (D) indices for knowledge scale items

Discrimination indices offer valuable information into the correlation between individual items and their overall role within the knowledge scale. Positive indices indicate the capacity of an item to differentiate effectively between participants with low and high levels of knowledge. It can be seen that eight items had very high discrimination with indices greater than 0.40. Five items from the knowledge scale had an average discriminatory power, with values between 0.14 and 0.32. Only two items had a low discrimination index, 0.06 and 0.02, respectively (Table 1). This distribution suggests that the majority of respondents were able to answer these items correctly.

**Table 1:** Difficulty (d) and discrimination (D) indices of knowledge scale items

Knowledge area	d	D
(13) – Definition of pregnancy-induced hypertension	0.66	0.51
(14) – Definition of preeclampsia	0.47	0.06
(15) – Clinical/lab signs of severe preeclampsia	0.58	0.23
(16) – Time interval for BP measurement in preeclampsia diagnosis	0.90	0.14
(17) – Laboratory tests for preeclampsia diagnosis	0.79	0.42
(18) – Definition of GD	0.99	0.02
(19) – Oral glucose tolerance test (OGTT) procedure	0.84	0.41
(20) – Glycemic ranges for GD diagnosis using the oral glucose tolerance test (OGTT)	0.60	0.23
(21) – Definition of prematurity, risk factors for preterm birth	0.38	0.25
(22) – Risk factors for preterm birth	0.69	0.32
(23) – Definition of extreme prematurity	0.80	0.66
(24) – Timing of GD onset during pregnancy	0.56	0.65
(25) – Risk factors for GD	0.70	0.77
(26) – Specific manifestations of GD	0.43	0.58
(27) – Definition of proteinuria	0.92	0.49

Based on the values of the two indices, it can be stated that the knowledge scale is balanced in terms of difficulty. It contains three items with a low degree of difficulty: (16), (18), and (27), three items with a high degree of difficulty: items (14), (21) and (26), with the remaining items showing moderate difficulty. The specialized literature recommends the use of items with a medium degree of difficulty in composing the scales for measuring the level of knowledge [21].

### Evaluation of difficulty (d) and discrimination (D) indices for attitude scale items

Data analysis indicated high difficulty index values for most items within this scale, as presented in Table 2. In the context of attitudes, a higher difficulty index may suggest that respondents could deduce the more desirable answer, possibly without genuine knowledge. As can be seen in the data presented in Table 2, the discrimination indices for the analyzed items presented positive values, with 12 items presenting values of 0.40 or higher, which signifies an adequate ability to correctly discriminate between items with a low level of correct attitudes and those with a high level of positive attitudes.

**Table 2:** Difficulty and discrimination indices of attitude scale items

Knowledge area	d	D
(28.1) – Preventing hypertension in pregnancy	0.89	0.40
(28.2) – Importance of blood pressure (BP) monitoring	0.99	0.24
(28.3) – Self-education for caring for patients with GD	0.95	0.40
(28.4) – Prevention of GD	0,92	0,41
(28.5) – Prevention of preeclampsia in primiparous women	0,80	0,57
(28.6) – Assesing HBP and GD risk in patient care	0,97	0,30
(28.7) – Caring for patients with HBP and proteinuria	0.96	0.46
(28.8) – Impact of GD on pregnant women and the fetuses	0.90	0.55
(28.9) – Role of M and ON in preventing HBP	0.88	0.56
(28.10) – Role of M and ON in preventing GD	0.98	0.39
(28.11) – Prioritizing care for pregnant women with HBP/GD	0.93	0.55
(28.12) – Recognizing HBP-induced discomfort in pregnant women	0.69	0.06
(28.13) – Recognizing the potential risk of the pregnant patient to develop HBP during pregnancy	0.82	0.19
(28.14) – GD severity	0.88	0.50
(28.15) – Need for continuous assessment of patients with GD/HPB	0.98	0.25
(28.16) – The impossibility of preventing GD	0.72	0.59
(28.17) – Prioritizing treatment of pregnant women with GD/HPB	0.96	0.46
(28.18) – Interest in HBP and GD prevention	0.95	0.64

### Evaluation of difficulty (d) and discrimination (D) indices for practice scale items

The difficulty indices for the practice scale items were generally high (Table 3), indicating a low difficulty level for the respondents. Most discrimination indices were good or very good, indicating that the items effectively differentiate between respondents with correct and incorrect practices.

**Table 3:** Difficulty and discrimination indices for practice scale items

Practice area	d	D
(29.1) – Educating patients with HBP about diet	0.74	0.46
(29.2) – Educating patients with GD about exercise	0.53	0.55
(29.3) – Educating expectant mothers about weight control	0.66	0.50
(29.4) – Educating patients about quitting alcohol/tobacco consumption	0,93	0,20
(29.5) – Educating patients with GD about dietary regimen	0,86	0,36
(29.6) – Redirecting patients with pregestational diabetes to the specialist consultation	0,84	0,39
(29.7) – Evaluating pregnant women for signs of HBP	0.76	0.48
(29.8) – Correct blood pressure measurement practices	0.68	0.61
(29.9) – Educating patients with HBP about dietary regimen	0.41	0.69
(29.10) – Educating patients about early signs of preeclampsia	0.82	0.43
(29.11) – Monitoring BP during postpartum visits	0.76	0.50
(29.12) – Educating patients about limiting anti-inflammatory medication	0.54	0.82
(29.13) – Educating patients with GD/HBP about future health risks	0.73	0.68
(29.14) – Assessing body mass index (BMI) during routine pregnancy evaluations	0.55	0.84
(29.15) – Recommending interdisciplinary consultations to control risk factors	0.57	0.84
(29.16) – Educating patients with BMI ≥ 30 about health risks	0.64	0.75
(29.17) – BP monitoring	0.85	0.39
(29.18) – Ensuring quality control of BP monitoring devices	0.87	0.25
(29.19) – Educating patients about self-collecting blood glucose	0.85	0.39
(29.20) – Using BP devices adapted to the size of the patient's arm	0.77	0.50
(29.21) – Educating patients about BP self-monitoring	0.85	0.43
(29.22) – Educating patients about constant blood glucose monitoring	0.86	0.39
(29.23) – Assessing patient understanding of BP self-monitoring	0.85	0.43

### Internal consistency of the evaluation scales

Cronbach's alpha coefficient was used to assess the internal consistency of the knowledge, attitude, and practice scales related to GD and HBP management. This coefficient measures how well the items within each scale relate to each other, with a higher alpha indicating greater consistency. A value of 0.70 is generally considered acceptable [22]. The analysis yielded a Cronbach's alpha of 0.729 (95% CI, 0.654–0.776) for the knowledge scale, exceeding the recommended threshold. Similarly, the attitude scale demonstrated a good alpha of 0.756 (95% CI, 0.690–0.814). The practice scale had a high alpha of 0.925 (95% CI, 0.905–0.943) (Table 4). These results indicate that the items within each scale effectively measured their respective constructs (knowledge, attitudes, practices). Consequently, all scales were retained in their original forms.

**Table 4:** Cronbach’s alpha coefficient for the three scales

Scale	Cronbach’s alpha coefficient	Confidence interval 95%
1. Knowledge scale	0.729	0.654 - 0.776
2. Attitudes scale	0.756	0.690 - 0.814
3. Practice scale	0.925	0.905 - 0.943

## DISCUSSION

This study aimed to develop and validate a reliable tool to assess the level of knowledge, attitudes, and practices of Romanian M and ON caring for pregnant patients with GD and gestational HBP. Although several researchers have developed studies on the knowledge, attitudes, and practices of M and ON, information on the validation of these tools is limited [6,13-16,19]. The results of our study demonstrate adequate psychometric qualities of the assessment tool. The questionnaire assessed a wide range of aspects related to the specific care given to patients with GD and gestational HBP by M and ON. Using the 3 evaluation scales included in the questionnaire, a comprehensive evaluation of the knowledge, attitudes, and practices of M and ON could be provided.

The analysis of responses across the three scales revealed good discrimination between participants with varying knowledge levels. Within the knowledge scale, item difficulties were well-distributed: three items were highly difficult, three had low difficulty, and nine had medium difficulty. Although the predominance of difficulty indices with increased values was observed for the attitude scale, the difficulty must be interpreted as a tendency of desirability. Basically, the higher the value of the difficulty indices, the more we can assume that the respondents could predict the socially desirable answer, which may not truly reflect their genuine attitudes or behaviors. Regarding the results obtained on the discrimination indices, the scale has an adequate discriminatory power that could differentiate between respondents with correct and less correct attitudes. Regarding the practice scale, the low difficulty indices suggest that participants were familiar with the described procedures. Furthermore, the uniform distribution of discrimination indices demonstrates the ability of the scale to effectively distinguish between respondents with correct and less correct practices.

The internal consistency analysis for each scale underscores the reliability of the questionnaire, as evidenced by the positive correlations between item scores and the overall scale scores. Specifically, the knowledge scale had a good Cronbach's alpha coefficient of 0.729 and 0.756, respectively, for the attitude scale. An excellent Cronbach’s alpha coefficient value of 0.925 was obtained for the scale of the practice.

Developing a valid and reliable assessment tool is the first step in identifying the educational needs of health professionals and designing individualized medical education plans based on identified priority training needs. Given its easy-to-use administration and scoring, the tool can be used in multiple educational activities, including midwifery practice or university training for M or ON. It can be successfully used for evaluating the impact of educational programs on the care of patients with GD and gestational HBP applied in two stages, before and after the completion of the training. Moreover, the study highlights the importance of using validated and reliable tools to measure knowledge, attitudes, and practices in research, given that validity and reliability ensure accuracy and consistency in the data collection.

We believe the research conducted in this validation study is of significant practical importance, especially for nursing leadership. By providing a valid and reliable tool, we can facilitate future research on this topic, identify the training needs of medical staff before and after training programs, and enhance the care midwives and obstetric nurses provide to patients with GD and HBP. The study has certain limitations, primarily due to the relatively small number of participants and the single-hospital setting. Although the study was conducted in a single hospital, the sample size encompassed all available M and ON, ensuring representativeness within that setting. However, to enhance the generalizability of the findings, future research should involve a larger and more diverse sample population, potentially across multiple specialized hospitals in Bucharest. Nevertheless, we believe that the validity testing of this tool is crucial for future research, even with the small sample size. The tests demonstrated that the tool accurately identified the aspects it intended to measure. This ensures the credibility and accuracy of the data collected, providing confidence in subsequent research results. By using tools that have been tested for psychometric properties, measurement errors can be avoided. Specific statistical tests allow us to identify and remove items from the tool that are irrelevant or lack the necessary validity to effectively capture the concept being studied. Consequently, obtaining a validated tool can ensure valid and reliable results, ensuring the quality and reliability of data collected in future studies.

The findings of our study hold significant implications for the practice of midwives and obstetric nurses in Romania, as this research represents the first effort to validate a tool designed to assess knowledge, attitudes, and practices concerning the care of pregnant patients with GD and HBP. Moreover, internationally, literature on this subject is scarce, underscoring the contribution of our work to the global body of knowledge.

## CONCLUSION

Developing a tool to assess the knowledge, attitudes, and practices of midwives and obstetric nurses caring for pregnant patients is instrumental in designing targeted educational programs focused on training needs. In addition, it is a particularly useful tool for nursing leadership in designing annual continuing medical education plans. The results of this research delivered a valid and reliable tool that can be used in research, educational settings, and even individual assessments to identify specific learning needs.

## Data Availability

Further data is available from the corresponding author upon reasonable request.
